# Glucagon‐Like Peptide‐1 Receptor Agonists in Multiple Sclerosis: A Topical Narrative Review

**DOI:** 10.1002/acn3.70547

**Published:** 2026-10-12

**Authors:** Audrey Y. Chang, Farrah J. Mateen

**Affiliations:** ^1^ Division of MS and Neuroimmunology, Davee Department of Neurology Northwestern University Chicago Illinois USA

**Keywords:** glucagon‐like peptide‐1 receptor agonist, multiple sclerosis, neuroprotection, obesity, weight loss

## Abstract

The glucagon‐like peptide‐1 (GLP‐1) receptor agonists (RAs) are a relatively new class of drugs that were initially approved by the US Food and Drug Administration to improve glycemic control in patients with type 2 diabetes mellitus and, subsequently, weight reduction in patients with obesity. Multiple sclerosis (MS) is commonly associated with comorbidities for which GLP‐1RAs are indicated. An increasing prevalence of comorbid chronic diseases has increased the number of people living with MS (pwMS) who are prescribed a GLP‐1RA, for indications of obesity, diabetes, obstructive sleep apnea, metabolic‐associated steatohepatitis, and/or chronic kidney disease. This review addresses the following key considerations: (1) the use of GLP‐1RAs for weight loss in pwMS and obesity, including considerations related to drug tolerability and safety; (2) the efficacy of the GLP‐1RAs for their approved non‐MS indications in pwMS; and (3) the potentially pleiotropic effects of the GLP‐1RAs on the MS disease course beyond their approved indications.

## 1 Introduction

The glucagon‐like peptide‐1 receptor agonists (GLP‐1RAs) are a relatively new class of drugs approved to treat type 2 diabetes mellitus (T2DM) and obesity. Their popularity has surged; an estimated 1 in every 8 Americans has now tried a GLP‐1RA [1]. Multiple sclerosis (MS) is commonly associated with comorbidities for which GLP‐1RAs are indicated. As patients and physicians seek to manage comorbid insulin resistance and obesity in MS, GLP‐1RAs are increasingly discussed and prescribed for people living with MS (pwMS). There is also interest in expanding the therapeutic potential of GLP‐1RAs for new disease indications, including MS. Current published research probes the safety, tolerability, and efficacy of GLP‐1RAs for approved indications in pwMS. GLP‐1RAs are also being investigated to expand the available treatments that attenuate the MS disease course, with the hope that the drug class may possess neuroprotective properties. The objectives of this review are to update and inform MS clinicians on the current knowledge and research on the GLP‐1RA class of drugs in pwMS.

## 2 Methods

This is a topical narrative review. A search was conducted using PubMed.gov and Google Scholar (scholar.google.com) to identify relevant studies to GLP1‐RAs and MS. The search period covered the years from GLP‐1RA development to present, January 1, 2005–September 17, 2026. Articles published in English or with at least an abstract in English were reviewed. While this is not a systematic review, our selection of articles from search results and reference lists adhered to PRISMA guidelines. The search strategy combined the following four search strings (MeSH terms in PubMed):

1. [(glucagon‐like peptide‐1) OR (glucagon‐like peptide‐1 receptor agonist) OR (exenatide) OR (semaglutide) OR (liraglutide) OR (tirzepatide) OR (lixisenatide) OR (dulaglutide) OR (NLY01)] AND [(multiple sclerosis) OR (demyelinating autoimmune disease)]
2. (multiple sclerosis) AND (experimental autoimmune encephalitis) AND [(glucagon‐like peptide‐1) OR (glucagon‐like peptide‐1 receptor agonist) OR (exenatide) OR (semaglutide) OR (liraglutide) OR (tirzepatide) OR (lixisenatide) OR (dulaglutide) OR (NLY01)]
3. [(multiple sclerosis) OR (demyelinating autoimmune disease)] AND [(obesity) OR (overweight)]
4. [(glucagon‐like peptide‐1) OR (glucagon‐like peptide‐1 receptor agonist) OR (exenatide) OR (semaglutide) OR (liraglutide) OR (tirzepatide) OR (lixisenatide) OR (dulaglutide) OR (NLY01)] AND (comorbidity)

### 2.1 GLP‐1 and GLP‐1RAs

Glucagon‐like peptide‐1 (GLP‐1) is an incretin hormone that is critical to achieve postprandial glucose control. Primarily synthesized by intestinal L cells and brainstem neurons, GLP‐1 is released in response to undigested nutrients entering the small intestine [2]. Contributing to the “ileal brake” phenomenon, GLP‐1 signaling, through the vagus nerve and central nervous system (CNS), delays gastric emptying, improves satiety, and promotes glucose‐dependent insulin release [2, 3]. GLP‐1RA drugs mimic the action of endogenous GLP‐1 to pharmacologically improve glycemic control. Exenatide became the first US Food and Drug Administration (FDA)‐approved GLP‐1RA, in 2005, for the disease indication of diabetes [2]. Exendin‐4 is an isolated peptide from Gila monster venom, which is structurally similar to human GLP‐1 but resistant to physiologic degradation by dipeptidyl peptidase (DPP‐4) in the bloodstream [2, 3]. Modeled after Exendin‐4, the successful synthetic design of Exenatide inspired the development of more potent, long‐acting, CNS‐penetrant, and multi‐target agonists specific to the GLP‐1 receptor (GLP‐1R) (Table 1) [3].

**TABLE 1:** Currently approved glucagon‐like peptide‐1 receptor agonists (GLP‐1RAs) in the USA.

Generic (trade) name [4]	Dosing frequency [4]	Indication [4, 5, 6, 7]
Injectable GLP‐1 agonists		
Exenatide (Byetta)^a^	Twice daily	T2DM
Exenatide (Bydureon)^a^	Once weekly	Chronic weight management
Dulaglutide (Trulicity)	Once weekly	T2DM and cardiovascular disease
Liraglutide (Victoza)	Once daily	T2DM and cardiovascular disease
Liraglutide (Saxenda)	Once daily	Chronic weight management
Lixisenatide (Adlyxin)^b^	Once daily	T2DM
Semaglutide (Ozempic)	Once weekly	T2DM, cardiovascular disease, and CKD
Semaglutide (Wegovy)	Once weekly	Chronic weight management, cardiovascular disease, and MASH
Injectable dual GLP‐1/GIP agonists		
Tirzepatide (Mounjaro)	Once weekly	T2DM
Tirzepatide (Zepbound)	Once weekly	Chronic weight management and OSA
Oral GLP‐1 agonists		
Semaglutide (Rybelsus)^c^	Once daily	T2DM and cardiovascular disease
Semaglutide (Ozempic pill)	Once daily	T2DM and cardiovascular disease
Semaglutide (Wegovy pill)	Once daily	Chronic weight management
Orforglipron (Foundayo)	Once daily	Chronic weight management
Injectable combination insulin and GLP‐1 agonists [8]		
Insulin glargine and lixisenatide (Soliqua)	Once daily	T2DM
Insulin degludec and liraglutide (Xultophy)	Once daily	T2DM

^a^ Byetta and Bydureon were discontinued by drug manufacturer AstraZeneca on October 28, 2024, and October 25, 2024, respectively [9].

^b^ As of 2023, Adlyxin is no longer available in the United States [10].

^c^ Rybelsus is being phased out in the United States by manufacturer Novo Nordisk starting May 4, 2026, for reformulation and remarketing as the Ozempic pill [11].

### 2.2 GLP‐1RAs for Weight Loss in MS

GLP‐1RAs have been approved and marketed as an effective pharmacological option in the treatment of obesity. Obesity is defined as a body mass index (BMI) greater than 30 kg/m^2^ [12]. There are important links between obesity and the MS disease course. An exhaustive review of MS and obesity is beyond the scope of our review. However, we highlight a few important lines of evidence that suggest obesity management should be prioritized in pwMS.

First, the prevalence of obesity in many studies is high. As examples, in the USA, 42% of newly diagnosed pwMS were found to be obese in a multi‐center, observational, and prospective cohort, the Multiple Sclerosis Partners Advancing Technology and Health Solutions (MS PATHS) study [13]. In another prospective study cohort of 130 pwMS with at least moderate disability, 56% had a waist circumference consistent with abdominal obesity [14].

Second, there may be important clinical outcomes in the MS disease course that are impacted by the presence of obesity. An incident population‐based case control study of 3249 people in the Swedish MS registry found that obesity at baseline is associated with faster MS disease progression as measured by time to Expanded Disability Status Scale (EDSS) score 3 or 4, more frequent T2 hyperintense demyelinating lesions on MRI, worse health‐related quality of life measures, and higher risk of cognitive worsening [15]. A study of the German National MS registry corroborated many of these findings [16]. A population‐based study leveraging anthropometric data and long‐term follow up of Norwegian residents found that high BMI in individuals 14 to 24 years of age was associated with consistent, increased MS susceptibility later in life in both male and female individuals [17]. Even increased waist circumference alone was associated with higher odds of severe versus mild disability (odds ratio (OR) = 1.47) in a study of the North American Research Committee on MS registry (NARCOMS) [18]. While explanations of the mechanisms linking obesity to MS incidence and progression are epidemiological, early theories for obesity‐mediated autoimmunity include interactions of adipose tissue dysregulation [19], decreased vitamin D bioavailability [20], altered peripheral estrogen signaling [21], amplification of Epstein–Barr virus infection immune response [22], and gut microbiota dysbiosis [23].

Since MS is a complex, multifactorial disease without fully known causation, obesity remains an identifiable risk factor that is modifiable, but is neither necessary nor sufficient to explain the disease incidence. Importantly, there is no current consensus on how the prevalence of obesity among pwMS compares to the general population as population‐based studies in many countries are not being done [24]. Predisposition to physical disability and decreased physical activity in pwMS may also contribute to the relationship between obesity and MS‐focused outcomes [24]. Further longitudinal research will allow all Bradford‐Hill criteria on causation in epidemiology to be assessed in full. However, addressing the risk factor of obesity is likely still appealing for pwMS. Considering GLP‐1RA efficacy in reducing body weight in initial trials for people with overweight or obesity [25], it is reasonable to assume that pwMS are interested in starting a GLP‐1RA to reduce body weight for a number of assumed health benefits. Taken together, effective treatment of comorbidities by GLP‐1RA may itself have disease‐modifying potential in MS (Table 2) [37].

**TABLE 2:** Glucagon‐like peptide‐1 receptor agonists (GLP‐1RAs) for US FDA‐approved and emerging indications in comorbidities of multiple sclerosis (MS).

Comorbidity	Relevance to MS
Approved indications	
Type 2 diabetes mellitus	T2DM is associated with MS, and poor glycemic control exacerbates cognitive impairment that is both symptomatic of MS and progresses with age [26, 27].
Obesity	Obesity is common in pwMS and is associated with elevated risk of MS disease onset and progression [13, 14, 15, 16, 17, 18, 19, 20, 21, 22, 23].
Cardiovascular disease	Cardiovascular disease incidence is elevated in pwMS, and myocardial infarction is the leading cause of mortality in MS [28].
Obstructive sleep apnea	OSA is associated with MS and shares symptoms of fatigue and cognitive deficits [29].
Metabolic‐associated steatohepatitis	MASH and MS share associations with obesity, and MS treatment can benefit from hepatoprotective agents for MS‐associated liver issues (e.g., drug‐induced liver injury from some DMTs and autoimmune hepatitis) [30, 31].
Chronic kidney disease	Elevated risk of neurogenic bladder and adverse effects from certain MS treatments put pwMS at increased risk of kidney‐related complications [32, 33, 34].
Emerging indications^a^	
Alcohol use disorder	Metabolic and reward‐related pathways may be shared between obesity and alcohol use disorder. Reduction of alcohol dependence and calorie consumption may have direct or indirect effects on mood and quality of life [35].
Osteoarthritis	Obesity increases the risk of both MS and osteoarthritis, exacerbating shared symptoms of pain, fatigue, and decreased mobility. The STEP 9 trial of 407 participants with obesity and knee osteoarthritis found semaglutide to result in significant reductions in both body weight and pain related to osteoarthritis [36].

^a^ _Ongoing clinical trials are investigating benefits of GLP‐1RAs in asthma_, _chronic obstructive pulmonary disease_, _inflammatory bowel disease_, _which demonstrate relevance to MS regarding exercise tolerance_, _fatigue_, _and microbiome dysbiosis_, _respectively_.

### 2.3 GLP‐1RAs for Approved Indications That Are Comorbidities in MS

Five percent of pwMS currently have comorbid T2DM [26]. However, as T2DM prevalence increases in the general population and with age, more pwMS may start a GLP‐1RA to improve blood sugar—the dysregulation of which can exacerbate the cognitive impairment that typically presents later in life [27]. Preventing neurological decline incurred by metabolic disease is protective for pwMS who are facing potentially compounding sources of worsened physical and cognitive disability.

Obstructive sleep apnea (OSA) is common in pwMS [29]. Treatment of OSA is relevant to MS clinicians to improve shared symptoms of fatigue. Tirzepatide is the first FDA‐approved medication and GLP‐1 to treat OSA in combination with diet and exercise, since 2024 [5]. OSA is strongly associated with obesity and causes hypertension and chronic intermittent hypoxia [38]. A Phase III clinical trial demonstrated that tirzepatide effectively helped reduce body weight, apnea frequency, hypoxic burden, blood pressure, and fatigue in patients with OSA [38]. A mechanistic study of another GLP‐1RA isolated liraglutide's ability to enhance Nrf2 translocation and downregulate nuclear factor kappa‐light‐chain‐enhancer of activated B cells (NF‐kB) signaling in in vitro models of chronic intermittent hypoxia [39]. By reducing body weight, blood pressure, and expression of neuroinflammatory factors in the setting of OSA, GLP‐1RAs may help to mitigate fatigue in pwMS. There are no specific reports of GLP1‐RAs in pwMS who have OSA.

Cardiovascular disease incidence is elevated in pwMS, and myocardial infarction is a leading cause of excess mortality in pwMS [28]. GLP‐1RAs improve ventricular function in the ischemic human heart in pre‐clinical and clinical studies [40]. The GLP‐1RA semaglutide was first approved by the FDA in 2024 to reduce the risk of life‐threatening cardiovascular events in adults with cardiovascular disease and obesity or overweight [6]. The ability of GLP‐1RAs to reduce cardiovascular risk in T2DM is well established in clinical trials [41]. Semaglutide has more recently been shown to reduce the risks of death from cardiovascular causes, nonfatal myocardial infarction, and nonfatal stroke in patients with overweight or obesity, even in the absence of diabetes [42]. While reduction in body weight and waist circumference reflect the known metabolic effects of GLP‐1RAs, cardioprotective effects of GLP‐1RAs have been demonstrated through improvements to cardiovascular outcomes in the absence of significant weight loss [43]. Ongoing clinical studies could elucidate whether targeting cardiovascular health via GLP‐1RAs improves vitality for pwMS [44].

GLP‐1RAs are also approved for treatment of metabolic‐associated steatohepatitis (MASH). MASH shares strong associations with obesity, where fat build‐up in the liver causes inflammation and scarring. In 2025, the FDA approved semaglutide to MASH in adults with moderate‐to‐advanced fibrosis [7]. Semaglutide demonstrated efficacy in a Phase III clinical trial, with improved resolution of steatohepatitis and reduction of liver fibrosis compared to placebo [30]. The hepatoprotective effects of GLP‐1RAs outside of MASH are contradictory. While MS patients could benefit from hepatoprotective effects, possibly in the rare case of drug‐induced liver injury from disease‐modifying therapies including pipeline agents like Bruton's tyrosine kinase inhibitors (BTKis), it is also notable that there is a rare, idiosyncratic incidence of hepatotoxic effects from GLP‐1RAs [31].

GLP‐1RAs are similarly approved for the treatment of chronic kidney disease in T2DM. GLP‐1RAs demonstrate renoprotective effects in chronic kidney disease, which has the risk factors of T2DM and obesity. In an international, randomized trial, semaglutide reduced the incidence of major kidney disease events [32]. While kidney disease is not commonly associated with MS, pwMS can experience kidney‐related complications due to an elevated risk of neurogenic bladder dysfunction and rarely, adverse effects from certain MS treatments, such as alemtuzumab [33]. The renoprotective potential of GLP‐1RAs is further complicated by rare but documented reports of kidney‐related adverse events, including acute kidney injury (AKI) and acute interstitial nephritis (AIN) [34]. The general paucity of data on hepatic and renal comorbidities in pwMS warrants further investigation.

### 2.4 GLP‐1RAs for Emerging Indications in People Living With MS

There are currently more than 2000 clinical trials conducted on GLP‐1RAs that are investigating novel applications of GLP‐1RAs across organ systems [45]. The ability of GLP‐1RAs to treat alcohol use disorder is an emerging consideration currently under investigation. Semaglutide is associated with a reduction in heavy drinking days in patients with alcohol use disorder and comorbid obesity [35]. GLP‐1 and GLP‐1RAs may modulate metabolism and brain reward pathways shared between obesity and alcohol use disorder. Semaglutide also improved secondary measures of self‐evaluated psychological health [35]. Depressive symptom reduction could be secondary to decreased alcohol dependence or a direct result of decreased inflammation and oxidative stress on mood. Current evidence is contradictory as to whether GLP‐1RAs are associated with increased or decreased levels of depression and anxiety [46, 47]. The association between alcohol consumption and disability in MS is also inconsistent [48]. However, ongoing clinical trials (NCT05895643 and NCT07559500) using mood and quality of life as secondary outcome measures to GLP‐1RA efficacy in alcohol use disorder may inform the treatment of depression and anxiety in MS complicated by alcohol dependence.

### 2.5 GLP‐1RAs: Data in People Living With MS

With the increasing popularity of GLP‐1RAs, studies are beginning to clarify the experiences of pwMS taking GLP‐1RAs. A small, single‐centered, retrospective study investigated the safety, tolerability, and efficacy of GLP‐1RAs in pwMS [49]. For 49 adult pwMS taking semaglutide or tirzepatide for an average of 24.2 months, the average weight loss was 0.47 kg/month. This value is approximately half of the weight loss documented in the initial Phase III clinical trial studying semaglutide in non‐MS patients with obesity [25]. While falling short of levels expected from non‐MS patients, GLP‐1RAs demonstrated efficacy in weight loss, considering confounding factors from MS disease and treatment, such as steroid use. In regard to tolerability, 29% of pwMS reported gastrointestinal side effects on the GLP‐1RAs; however, only 6% discontinued the GLP‐1RA due to intolerability, providing initial reassurance that pwMS do not have an increased risk of adverse reaction to GLP‐1RAs. MS disease activity was measured with one patient experiencing a clinical relapse and four patients with new subclinical lesions on MRI while taking GLP‐1RAs. Given that 24% of patients were not taking a DMT, the authors concluded that MS disease outcomes fall within an expected range and appeared safe for use by pwMS in this small cohort.

A similar retrospective study of 60 pwMS with a short follow‐up period of 3.0 ± 3.8 years corroborated these trends, adding serum 25‐hydroxy‐vitamin D levels as a measure of efficacy [50]. Lower serum vitamin D levels have been associated with an increased MS disease relapse risk and new incident T2 hyperintense brain lesions on MRI in pwMS [51]. Following GLP‐1 medication initiation, the study cohort had a decrease in average BMI and an increase in vitamin D values without progression in EDSS scores or timed 25‐ft walk (T25FW) duration, suggesting that GLP‐1RAs are effective for promoting weight loss and increased vitamin D bioavailability. While the investigators were unable to capture long‐term effects of GLP‐1RAs in pwMS, these studies provide early reassurance for MS neurologists and patients. No unexpected findings of adverse reactions for patients with MS compared to the general population or increased risk of MS disease progression were found. Further study is required to investigate whether stability of MS disease can be attributable to GLP‐1RA administration through longer‐term and larger‐scale studies. This is particularly true when evaluating retrospective data from the earliest years of GLP‐1RA availability, since the use of GLP‐1RAs by pwMS has now expanded beyond its initial FDA‐approved indication of T2DM.

### 2.6 Prescribing Considerations of GLP‐1RAs

An initial consideration is cost to pwMS who may already have co‐payment requirements for a DMT and medications for symptom control. For many patients, GLP‐1RAs are particularly expensive. Only 1%–2% of eligible patients in the USA are prescribed a medication or offered surgery to assist with obesity treatment [52]. The 2024 KFF Health Tracking Poll revealed that more than 50% of adults who have taken GLP‐1RAs say the cost was difficult to afford, including 22% who said it was “very difficult.” [1] Uptake of GLP‐1RAs for patients with and without MS is limited by eligibility requirements, high costs with varied insurance coverage, and hesitations from both patients and prescribers. A cross‐sectional survey of the self‐reported NARCOMS Registry found that among 4181 pwMS, although 40% of individuals had an indication, only 14.6% had ever taken a GLP‐1RA [53].

An emerging area of caution in pwMS taking GLP1‐RAs is sarcopenia, given that skeletal muscles possess GLP‐1Rs. Considering that GLP‐1RAs can induce weight loss close to the magnitude achieved by bariatric surgery, there is clinical concern about the potential adverse effects on lean muscle mass [54]. PwMS are already at risk for sarcopenia. One study found one‐fifth of pwMS have sarcopenia, which is associated with a high‐degree of fatigue and an inability to exercise [55]. Loss of temporal muscle thickness is accelerated in MS and correlated with disability [56]. Current studies display heterogeneity in reported effects of different GLP‐1RAs on skeletal muscle changes. In the STEP‐1 trial of semaglutide, the fraction of weight loss from lean mass was 45.2% [25, 57]. In the SURMOUNT‐1 study of tirzepatide, the fraction of weight loss from lean mass was 25.7% [57, 58]. Conversely, limited preclinical and clinical data suggest GLP‐1RAs can improve skeletal muscle health [59]. Further study is warranted to evaluate the therapeutic cost and benefit in pwMS receiving GLP‐1RAs and the most at‐risk subgroups.

Another uncertain effect of GLP‐1RAs relevant to pwMS is nonarteritic anterior ischemic optic neuropathy (NAION). NAION is the most common form of acute optic nerve injury in adults older than 50 years old [60]. One retrospective study, from the Massachusetts Eye and Ear Infirmary, first quantified their anecdotal experience of an association between semaglutide and NAION [61]. In 16,827 patients who were overweight or obese, incidence of NAION in the semaglutide‐treated group was 6.7% versus 0.8% in the non‐treated group [61]. Subsequent retrospective studies found a more modest increase of NAION associated with semaglutide use [62]. One such study found the absolute NAION risk with GLP‐1RA treatment to be 0.09%, corresponding to a 0.037% risk difference [63]. Causality cannot yet be confirmed, but caution may be warranted for patients with pre‐existing risk factors. While a crowded optic disc is the primary risk factor, pwMS share common vascular risk factors of NAION, including diabetes, cardiovascular disease, and OSA [60]. As pwMS are already at increased risk of visual loss from the sequelae of optic neuritis, further ophthalmic complications are especially problematic for pwMS and should be weighed accordingly. Since none of these studies have been population‐based, all estimates of NAION are subject to referral and other forms of bias.

Finally, it is notable that the major tolerability concerns for GLP‐1RAs are nausea and gastrointestinal upset. Although MS does not have specific vulnerability for these intolerances, pwMS may conflate intolerance of GLP‐1RAs for intolerance of their other medications. In the case of the fumarate class of MS treatment, nausea and gastrointestinal symptoms may be difficult to disaggregate. Similarly, GLP‐1RAs may add to the needle or pill burden for patients already taking multiple medications. It is not uncommon for patients to have periods of discontinuation on the GLP‐1RAs when tolerability lowers, leading to difficulties in measurement of their efficacy. It would be important that a DMT is not stopped at the same time, for example, a sphingosine 1‐phosphate inhibitor, if intolerabilities are difficult to attribute by patients to one medication versus another.

### 2.7 GLP‐1RAs and Neuroprotection

GLP‐1RAs are putatively anti‐inflammatory in various models of chronic disease [64]. Inflammatory markers such as C‐reactive protein (CRP) and tumor necrosis factor alpha (TNF‐α) and various interleukins can be reduced with GLP‐1RA administration in T2DM, cardiovascular disease, obesity, and kidney disease [64]. GLP‐1RA action is commonly associated with the NF‐kB signaling pathway to reduce systemic and local inflammation [64]. GLP‐1Rs are present in the CNS, particularly brain areas that are essential for cognition [65], suggesting a potential therapeutic target in neurological disorders by a similar mechanism.

By comparison, the potential for neuroprotective effects has been studied in people with Parkinson's disease (PD). T2DM increases the risk and rate of PD progression [66]. GLP‐1RAs are of interest to PD clinicians as an anti‐diabetic medication with potential neuroprotective effects. The ability of GLP‐1RAs to modulate neuroinflammation has been assessed in pre‐clinical and clinical studies. NLY01 is an experimental PEGylated form of exendin‐4 that is used in studies for its brain‐penetrant and long‐acting properties [67]. In PD mouse models, NLY01 prevents microglial‐mediated conversion of astrocytes to a neurotoxic phenotype [67]. Blockade of neurotoxic astrocyte conversion protects against dopaminergic neuronal loss and behavioral deficits characteristic of PD [67]. Other clinical studies also suggest the possible neuroprotective potential of GLP‐1RAs in PD. A retrospective study from a global collaborative database found a statistically significant risk reduction for PD in obese patients treated with semaglutide (relative risk (RR) = 0.574) [68]. In a Phase II, randomized trial, lixisenatide treatment resulted in less progression of motor disability than placebo but was associated with gastrointestinal side effects [69]. Sustained benefit in the off‐medication state may be suggestive of neuroprotective mechanism with direct effects on CNS.

However, a clinical trial investigating NLY01 at 2.5 and 5.0 mg was not associated with any improvement in PD motor or non‐motor features [70]. While GLP‐1RAs have been observed to cross the blood–brain‐barrier (BBB) in pre‐clinical studies [71, 72, 73], the extent and clinical translation of GLP‐1 activation of neural targets remain inconclusive and represent an unresolved research gap in neuroprotection theories. An ongoing Phase II trial is assessing the effects of semaglutide on PD‐associated parameters, with blood and cerebrospinal fluid sample analysis to confirm the penetration of semaglutide across the BBB and assess its ability to modulate disease progression [74]. Association between CNS occupancy and therapeutic outcomes of GLP‐1RAs remains to be proven in neurological disorders.

### 2.8 GLP‐1RAs: Mechanism of Action in MS

The potential therapeutic action of GLP‐1RAs in MS is informed by ongoing experimentation using animal models of MS. Experimental autoimmune encephalomyelitis (EAE) in mice is an animal model used for relapsing–remitting MS. Inducing EAE involves triggering an immune response against myelin protein, which mimics the CNS inflammation of MS in humans. In preclinical studies, expression of GLP‐1R in the CNS is not well elucidated and may vary between species and technique of analysis [75]. Studies using EAE‐induced mice have suggested that GLP‐1RA exerts anti‐inflammatory and antioxidant effects relevant to MS through immune cell modification and cAMP/PKA and PI3K/Akt pathways [76, 77, 78, 79, 80]. While EAE markedly downregulates GLP‐1 and GLP‐1R, GLP‐1RA administration rescues GLP‐1R signaling. Studies have demonstrated this supplantation of GLP‐1RA in EAE models to improve clinical symptoms, amend behavioral test performance, maintain CNS organ weight, reduce spinal cord damage, and attenuate reduction of myelin staining in EAE [76, 77, 78, 79, 80]. Exendin‐4 administration reduced mRNA expression of pro‐inflammatory cytokines that are upregulated in EAE‐activated microglia via the NF‐kB pathway [76]. In rodent models, liraglutide delayed onset and reduced severity of EAE by upregulating Nrf2 and mitochondrial antioxidant MnSOD and reducing the neurodegenerative marker amyloid precursor protein in the brain [77]. Replicated in mice, liraglutide ameliorated demyelination and inflammation, possibly through regulation of pAMPK, autophagy influx, pyroptosis‐related NLRP3 pathway [79], and splenic helper T cells [78]. Semaglutide increased CREB/BDNF/MBP protein, markers of myelination, while reducing NF‐kB and TNF‐α [80].

However, the myelin‐modulating potential and mechanism of action of GLP‐1RA have so far elicited mixed results. A study using a cuprizone‐induced mouse model of acute demyelination suggested a remyelinating property of GLP‐1RA [81]. In *Cup* mice, liraglutide restored myelinated nerve staining, which translated to an improvement in behavior and brain weight [81]. Mechanistically, liraglutide elevated p‐AMPK/SRT1 levels, MBP and Olig2 protein expression, and autophagy biomarkers, while decreasing microgliosis and pro‐inflammatory cascades [81]. However, a subsequent study challenged these findings by identifying that the anorexigenic effect of GLP‐1RA confounds the amount of cuprizone ingested in mouse models [82]. By controlling for cuprizone and adding a myelin‐specific immune response to the *Cup* model, this study found that NLY01 had no effect on demyelination or remyelination in the setting of inflammatory barriers specific to MS [82]. Given contradictory findings between GLP‐1RA and animal model types, the limitations of animal models to reflect drug mechanisms in MS may arise from heterogeneity in study design and approaches. The demyelinating and remyelinating potential of GLP‐1RA remains uncertain but warrants further clinical investigation to evaluate its therapeutic potential.

### 2.9 GLP‐1RAs: Future Indications Relevant to MS

Putative neuroprotective effects of GLP‐1RAs are currently supported by preclinical models, whereas human MS‐specific evidence remains limited, consisting of the two small retrospective cohorts previously discussed. Two ongoing trials are identified relevant to pwMS and GLP‐1RAs. The authors are part of an actively enrolling clinical trial at Northwestern University in Chicago, IL (NCT07207148) that is investigating long‐term outcomes of pwMS co‐treated with Ocrelizumab and GLP‐1RA. Measuring patient‐reported outcome measures (PROMs), clinical assessments, and biomarkers of disease, the study follows patients for 72 weeks each and will expand the evidence on co‐treatment of MS with Ocrelizumab and GLP‐1RAs to inform patients and prescribers about longer‐term safety, tolerability, and disease‐modifying potential of GLP‐1RAs in MS treatment. A second ongoing clinical trial at Johns Hopkins University in Baltimore, MD (NCT07497399) similarly aims to evaluate long‐term outcomes of GLP‐1RA treatment for pwMS. This nearly 2‐year study implements the novel GLP‐1RA NLY01, which has demonstrated promise in clinical trials for PD patients to reduce inflammation. Measuring change in normalized brain parenchymal volume (nBPV) through MRI, researchers will evaluate the extent of GLP‐1RA efficacy and consider possible mechanisms of neuroprotection in pwMS.

The complex nature of the MS disease course makes it unlikely that any standalone therapy will demonstrate dramatic efficacy on MS disease progression. However, ongoing clinical trials of GLP‐1RAs may suggest the capacity for GLP‐1RAs to contribute to disease‐modifying potential as part of an MS treatment regimen. For example, nonactive secondary progressive MS overall lacks effective disease‐modifying treatments. Statins are a medication class that is efficacious and well‐tolerated in the treatment of hyperlipidemia and prevention of cardiovascular and cerebrovascular disease [83]. In a randomized controlled trial of 140 pwMS, high‐dose simvastatin was associated with a 43% reduction in the annualized rate of whole‐brain atrophy, with no safety or tolerability difference to placebo [83]. Considering the inability of simvastatin to modulate inflammatory markers, GLP‐1RAs could be of interest as a combination therapy along with statins to achieve a higher likelihood of clinically recognizable disease‐modifying potential for pwMS.

For people with obesity, systematic reviews have found the effects of exercise on psychological outcomes secondary to weight loss inconclusive [84, 85]. In the general population, exercise demonstrates an inverse relationship with depression [84]; however, pwMS and obesity are often disproportionately burdened by physical, cognitive, and psychological symptoms and require more MS‐focused research. A retrospective study found weight‐reducing pharmacological and surgical treatments in people with obesity to be associated with improved quality of life and psychological functioning [86]. Improvement of mood with GLP‐1RA use may originate from weight reduction itself [86]. A clinical trial [87] randomized 71 pwMS and obesity to a weight loss intervention of calorie reduction and increased physical activity: two‐thirds of participants in the treatment condition experienced clinically significant weight loss, which was associated with improved mobility, endurance, and quality of life [87]. For pwMS and obesity, GLP‐1RA initiation, supplemental to routine physical activity and dietary modification, may improve feasibility of clinically significant, sustainable weight loss with secondary improvements to physical and psychological wellbeing in MS.

## 3 Limitations

As a topical narrative review, our work possesses inherent limitations. In the absence of a systematic, reproducible protocol utilized in systematic reviews, there is the potential for selection bias. However, we made committed efforts to provide an objective and comprehensive review of current literature.

## 4 Conclusions

The GLP‐1RAs represent an exciting therapeutic drug class for pwMS, both for the treatment of comorbidities and for the potential to act as specifically beneficial treatment in MS. The GLP1‐RAs are an explosive, multi‐billion‐dollar industry, and it behooves the MS community to study this drug class, often prescribed directly to patients through third party telehealth, for their safety, tolerability and efficacy. Moreover, as drug cost barriers are ameliorated, uptake could further increase. Multiple other GLP‐1RAs are in the therapeutic pipeline. Selection from among them for tolerability, such as injection reactions, and safety, such as any risk of optic neuropathy, will be of high importance in the MS community. At least 15% of people are clinically obese at the time of MS diagnosis and weight loss itself is a specific risk factor modification that can improve the outcome of obese pwMS [16]. Weight loss interventions of calorie reduction and increased physical activity can be difficult to implement for pwMS, particularly with symptoms of fatigue, imbalance, motor weakness, and sensory changes. By pharmacologically inducing weight loss and potentially reducing weight quickly, GLP‐1RAs may promote return to regular aerobic physical activity for pwMS, leading to secondary benefits on mood and energy levels. While early observational data of GLP‐1RAs is reassuring, available evidence does not yet establish disease‐modifying efficacy for this class of drugs. Prospective randomized control trials are needed to fully establish the roles of the GLP‐1RAs in pwMS.

## Author Contributions

Audrey Y. Chang contributed to study design, drafting, writing, and editing of the manuscript. Farrah J. Mateen contributed to study design, editing, and supervising of the manuscript.

## Funding

The authors have nothing to report.

## Conflicts of Interest

The authors are conducting a clinical trial on GLP‐1RAs in people with MS at Northwestern University, funded through an investigator‐initiated study (PI: Mateen) from Genentech Inc. The funder of the trial had no role in the design, conduct, writing, editing, or decision to submit this manuscript for publication.

## Data Availability

Data sharing not applicable to this article as no datasets were generated or analysed during the current study.
